# Aggregability of the SQSTM1/p62-based aggresome-like induced structures determines the sensitivity to parthanatos

**DOI:** 10.1038/s41420-024-01838-2

**Published:** 2024-02-12

**Authors:** Shuhei Hamano, Takuya Noguchi, Yukino Asai, Ryo Ito, Ryuto Komatsu, Tetsu Sato, Aya Inoue, Tomoe Maruyama, Tada-aki Kudo, Yusuke Hirata, Sawako Shindo, Yasuo Uchida, Gi-Wook Hwang, Atsushi Matsuzawa

**Affiliations:** 1 Laboratory of Health Chemistry, Graduate School of Pharmaceutical Sciences, Tohoku University, Sendai, Japan; 2 Division of Membrane Transport and Drug Targeting, Graduate School of Pharmaceutical Sciences, Tohoku University, Sendai, Japan; 3 Division of Oral Physiology, Graduate School of Dentistry, Tohoku University, Sendai, Japan; 4 Laboratory of Environmental and Health Sciences, Faculty of Pharmaceutical Sciences, Tohoku Medical and Pharmaceutical University, Sendai, Japan; 5 Department of Environmental Toxicology, Meiji Pharmaceutical University, Tokyo, Japan; 6 Department of Molecular Systems Pharmaceutics, Graduate School of Biomedical and Health Sciences, Hiroshima University, Hiroshima, Japan

**Keywords:** Cell death, Post-translational modifications

## Abstract

Overactivation of poly (ADP-ribose) polymerase-1 (PARP-1) triggers a noncanonical form of programmed cell death (PCD) called parthanatos, yet the mechanisms of its induction are not fully understood. We have recently demonstrated that the aggresome-like induced structures (ALIS) composed of the autophagy receptor SQSTM1/p62 and K48-linked polyubiquitinated proteins (p62-based ALIS) mediate parthanatos. In this study, we identified the D1 dopamine receptor agonist YM435 as a unique parthanatos inhibitor that acts as the disaggregating agent for the p62-based ALIS. We found that YM435 structurally reduces aggregability of the ALIS, and then increases its hydrophilicity and liquidity, which prevents parthanatos. Moreover, dopamine and L-DOPA, a dopamine precursor, also prevented parthanatos by reducing the aggregability of the ALIS. Together, these observations suggest that aggregability of the p62-based ALIS determines the sensitivity to parthanatos, and the pharmacological properties of YM435 that reduces the aggregability may be suitable for therapeutic drugs for parthanatos-related diseases such as neurodegenerative diseases.

## Introduction

Poly (ADP-ribose) polymerase-1 (PARP-1) is a key stress-responsive enzyme that mainly contributes to the maintenance of genomic stability and DNA repair [1]. In particular, PARP-1 functions as a sensor of DNA single-strand breaks (SSBs) that is the most frequent genomic damage [1]. Thereafter, PARP-1 promotes DNA repair by promoting poly-ADP-ribosylation of DNA repair factors [1]. On the other hand, PARP-1 triggers proinflammatory responses and cell death under pathological conditions [2–4]. Therefore, PARP-1 is a multifunctional protein that mediates a wide variety of stress responses. Notably, when the overactivation of PARP-1 is caused by pathologic stresses, PARP-1 overproduces poly (ADP-ribose) (PAR), which promotes the release of PAR into the cytoplasm. PAR released into the cytoplasm binds to the mitochondrial-localized protein apoptosis-inducing factor (AIF), and thereby promotes its nuclear translocation triggering parthanatos [1]. Parthanatos has been implicated in the development and progression of various diseases, including neurodegenerative diseases [5–9], ischemia-reperfusion injury [10, 11], diabetes [12], and age-related macular degeneration [13]. Thus, parthanatos has attracted attention as an attractive therapeutic target in these diseases.

Neurodegenerative diseases, such as Alzheimer’s disease, Parkinson’s disease, amyotrophic lateral sclerosis (ALS), are diseases in which neuronal function declines due to loss or death of nerve cells, leading to various neurological disorders [14]. Since aging is a risk factor of the neurodegenerative diseases, the number of patients accompanying aging population is expected to increase explosively [15]. However, no effective therapeutic treatment has been established to prevent the progression of neurological dysfunction, and therefore its development is an urgent issue in modern medicine. As mentioned above, accumulating evidence indicates that parthanatos is involved in the neurologic deficits in the neurodegenerative diseases [5–9]. In particular, protein aggregates responsible for triggering neurodegenerative diseases (e.g., aggregation of amyloid β, α-synuclein, and TDP-43) initiate parthanatos through oxidative stress. Therefore, parthanatos has emerged as an attractive therapeutic target for the neurodegenerative diseases.

Our cellular experiments have demonstrated that protein misfolding caused by oxidative stress promotes the formation of the p62-based ALIS composed of the autophagy receptor SQSTM1/p62 and K48-linked polyubiquitinated proteins, which triggers parthanatos [16]. Although molecular mechanisms by which the p62-based ALIS are involved in the PARP-1-dependent cell death machinery are still unclear, poly-ADP-ribosylation (PARylation) of the p62-based ALIS mediated by PARP-1 may play a key role in parthanatos (unpublished data). On the other hand, we have recently demonstrated that supersulfides dissolve the p62-based ALIS, and thereby inhibited subsequent parthanatos [17]. Mechanistically, the supersulfide donor Na_2_S_4_ promotes the persulfidation of heat shock protein 90 (HSP90) at Cys521, leading to the activation of heat shock factor 1 (HSF1). The activated HSF1 transcriptionally upregulated HSP70, which promotes disaggregation of the p62-based ALIS [18]. Therefore, the stability of the p62-based ALIS seems to determine the sensitivity to parthanatos. In addition, considering that p62 is included in the neurodegenerative disease-related aggregates, such as Levy’s body, Huntington’s aggregate, and in the neurofibrillary degeneration in Alzheimer’s disease, targeting the p62-related aggregates may be effective for the treatment of neurodegenerative diseases [19–23].

It has been reported that various compounds containing the catechol skeleton, such as polyphenols and catecholamines, can suppress the protein aggregate formation in both in vitro and in vivo [24–26]. Although the detailed mechanisms by which these compounds dissolve the protein aggregates are still unknown, compounds, such as polyphenols, that act as both antioxidants and disaggregating agent may be highly effective for the treatment of the neurodegenerative diseases. YM435, a small-molecule compound that has catechol skeleton, has been reported as a selective agonist of the D1 dopamine receptor [27]. By acting as a D1 dopamine receptor agonist, YM435 promotes vasodilatory effects and exocrine pancreas [27–30], and has been found to improve renal and heart failure at least in model animals [31–34]. In this study, we newly found that YM435 acts as both the antioxidant and disaggregating agent, and strongly inhibits parthanatos. Thus, our findings may provide insight into the novel therapeutic approaches for neurodegenerative diseases.

## Results

### YM435 suppresses oxidative stress-induced parthanatos by acting as an antioxidant

**Fig. 1:**
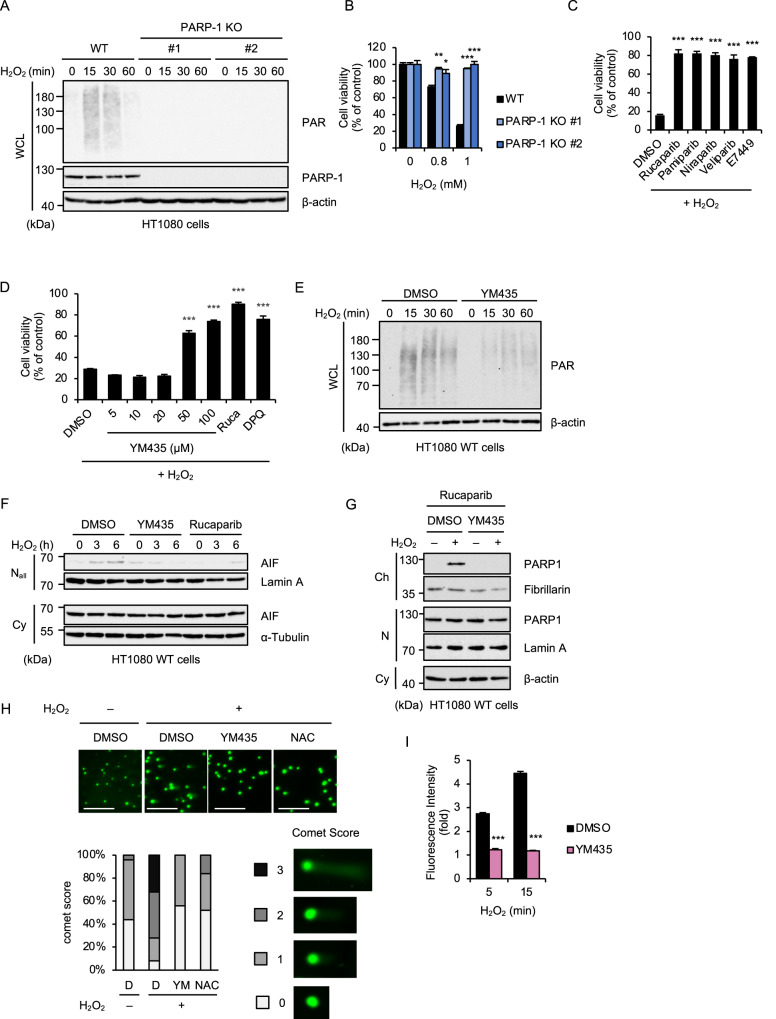
YM435 suppresses oxidative stress-induced parthanatos by acting as an antioxidant. **A** WT and PARP-1 KO HT1080 cells were treated with H_2_O_2_ (1.0 mM) for the indicated periods. Cell lysates were subjected to immunoblotting with the indicated antibodies. **B** WT and PARP-1 KO HT1080 cells were treated with the indicated concentrations of H_2_O_2_ for 5 h. Cell viability was measured by PMS/MTS assay. Data shown are the mean ± SD (*n* = 3). Statistical significance was tested by one-way ANOVA, followed by Tukey–Kramer test; ***p < 0.001, **p < 0.01, *p < 0.05 (vs. WT). **C** HT1080 cells were pretreated with Rucaparib (1 μM), Pamiparib (0.1 μM), Niraparib (1 μM), Veliparib (1 μM), or E7449 (1 μM) for 1 h and then treated with H_2_O_2_ (0.8 mM) for 5 h. Cell viability was measured by PMS/MTS assay. Data shown are the mean ± SD (*n* = 3). Statistical significance was tested by one-way ANOVA, followed by Tukey–Kramer test; *** *p* < 0.001 (vs. DMSO). **D** HT1080 cells were pretreated with the indicated concentrations of YM435, Rucaparib (1 μM), or DPQ (30 μM) for 1 h and then treated with H_2_O_2_ (0.8 mM) for 5 h. Cell viability was measured by PMS/MTS assay. Data shown are the mean ± SD (*n* = 3). Statistical significance was tested by one-way ANOVA, followed by Tukey–Kramer test; ***p < 0.001 (vs. DMSO). **E** HT1080 cells were pretreated with YM435 (50 μM) for 1 h and then treated with H_2_O_2_ (1.0 mM) for the indicated periods. Cell lysates were subjected to immunoblotting with the indicated antibodies. **F** HT1080 cells were pretreated with YM435 (50 μM), Rucaparib (1 μM) for 1 h and then treated with H_2_O_2_ (0.8 mM) for the indicated periods. The nuclear and cytoplasm extracts were subjected to immunoblotting with the indicated antibodies. **G** HT1080 cells were pretreated with YM435 (50 μM), Rucaparib (1 μM) for 1 h, and then treated with H_2_O_2_ (1.0 mM) for the indicated periods. The chromatin, nuclear, and cytoplasm extracts were subjected to immunoblotting with the indicated antibodies. (**H**) HT1080 cells were pretreated with YM435 (50 μM), NAC (2 mM) for 1 h, and then treated with H_2_O_2_ (1.0 mM) for 10 min (Scale bar, 100 μm). Comet scores were calculated according to comet tail length (*n* = 25). **I** HT1080 cells were pretreated with YM435 (50 μM), DCFH-DA (10 μM) for 1 h and then treated with H_2_O_2_ (1.0 mM) for the indicated periods. Quantification of ROS was calculated by detecting the fluorescence intensity of DCFH-DA. Data shown are the mean ± SD (*n* = 3). Statistical significance was tested by one-way ANOVA, followed by Tukey–Kramer test; ∗∗∗*p* < 0.001 (vs. DMSO). All data are representative of at least three independent experiments.

We have previously demonstrated that hydrogen peroxide (H_2_O_2_) preferentially initiates oxidative stress-induced parthanatos in the human fibrosarcoma HT1080 cell line [16]. As shown in Fig. 1A, H_2_O_2_ causes PARP1-dependent poly-ADP-ribosylation (PARylation), showing that H_2_O_2_ stimulates the PARP-1 activation. Reduced cell viability upon H_2_O_2_ treatment was completely restored in PARP-1 knockout (KO) HT1080 cells (Fig. 1B), or by the treatment with the conventional PARP-1 inhibitors (Fig. 1C). Collectively, these data confirm that H_2_O_2_ induces parthanatos in HT1080 cells. Interestingly, we newly found that YM435 inhibited the reduction of cell viability induced by H_2_O_2_ (Fig. 1D), and therefore examined whether YM435 inhibits parthanatos. As shown in Fig. 1E and F, YM435 inhibited the PARP-1 activation, and the nuclear translocation of AIF triggering large-scale DNA fragmentation, an index of the parthanatos induction, triggered by H_2_O_2_. YM435 also inhibited the accumulation of PARP-1 in the chromatin fraction required for parthanatos induction (Fig. 1G) [35, 36]. Moreover, comet assay revealed that YM435 inhibits oxidative stress-induced DNA damage, when comet scores were calculated as previously described (Fig. 1H) [37]. YM435 also inhibited the ROS accumulation (Figs. 1H and 1I). Therefore, these observations suggest that YM435 suppresses oxidative stress-induced parthanatos by acting as an antioxidant.

### Antioxidant activity of YM435 is effective in ex vivo

**Fig. 2:**
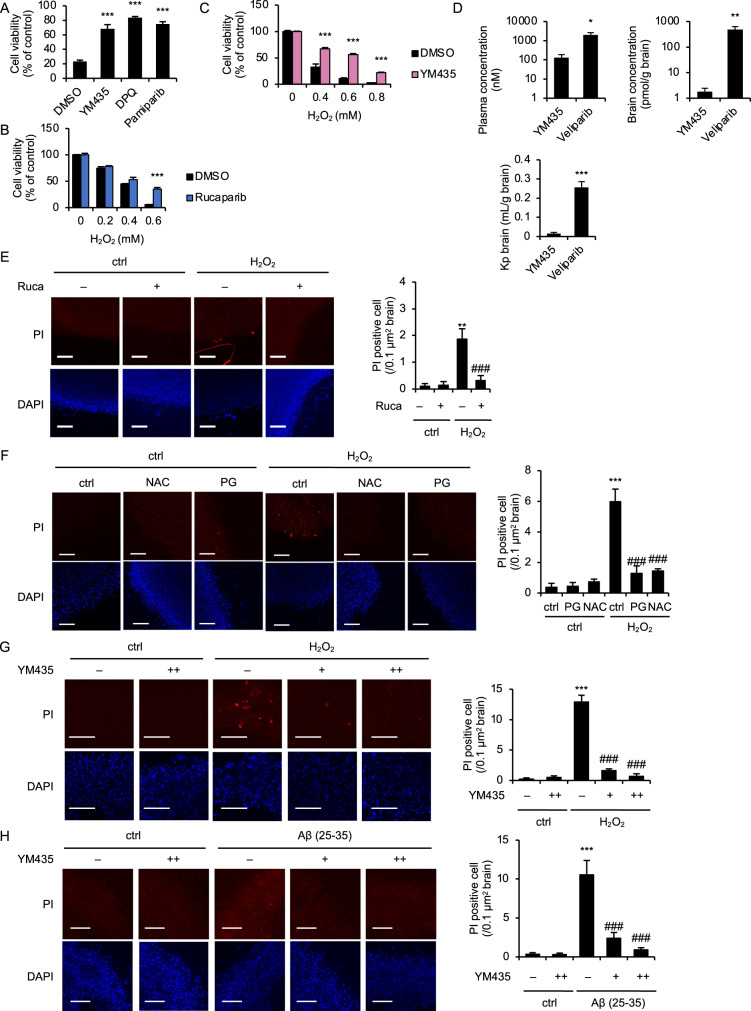
Antioxidant activity of YM435 is effective in ex vivo. **A** SH-SY5Y cells were pretreated with YM435 (40 μM), DPQ (30 μM), or pamiparib (0.5 μM) for 1 h, and then treated with H_2_O_2_ (0.6 mM) for 4 h. Cell viability was measured by PMS/MTS assay. Data shown are the mean ± SD (*n* = 3). Statistical significance was tested by one-way ANOVA, followed by Tukey–Kramer test; ***p < 0.001 (vs. DMSO). **B** PC12 cells were pretreated with Rucaparib (1 μM) for 1 h, and then treated with H_2_O_2_ (0.6 mM) for 6 h. Cell viability was measured by PMS/MTS assay. Data shown are the mean ± SD (*n* = 3). Statistical significance was tested by one-way ANOVA, followed by Tukey–Kramer test; ***p < 0.001 (vs. DMSO). **C** PC12 cells were pretreated with YM435 (50 μM) for 1 h, and then treated with the indicated concentrations of H_2_O_2_ for 6 h. Cell viability was measured by PMS/MTS assay. Data shown are the mean ± SD (*n* = 3). Statistical significance was tested by one-way ANOVA, followed by Tukey–Kramer test; ***p < 0.001 (vs. DMSO). **D** Plasma and brain concentrations of YM435 and veliparib, and Kp brain were quantified. Graphs are shown the mean ± SD (*n* = 4). Statistical significance was tested by Student’s t-test; *p < 0.05, **p < 0.01, ***p < 0.001 (vs. YM435). **E** Cultured brain slice was pretreated with Rucaparib (5 μM) for 24 h, and then treated with H_2_O_2_ (0.6 mM) for 6 h. Cell death was measured by PI staining (red) with 4’,6-diamidino-2-phenylindole (DAPI) nuclear staining (Scale bar, 100 μm). Quantification of propidium iodide imaging. Data shown are the mean ± SD (*n* = 5). Statistical significance was tested by one-way ANOVA, followed by Tukey–Kramer test; **p < 0.01 (vs. ctrl), ###*p* < 0.001 (vs. DMSO). **F** Cultured brain slice was pretreated with NAC (2 mM) and propyl gallate (PG; 40 μM) for 24 h, and then treated with H_2_O_2_ (0.6 mM) for 6 h. Cell death was measured by PI staining (red) with DAPI nuclear staining (Scale bar, 100 μm). Quantification of propidium iodide imaging. Data shown are the mean ± SD (*n* = 5). Statistical significance was tested by one-way ANOVA, followed by Tukey–Kramer test; ***p < 0.001 [vs. ctrl (–)], ###*p* < 0.001 [vs. ctrl (+)]. **G** Cultured brain slice was pretreated with the indicated concentrations of YM435 for 24 h, and then treated with H_2_O_2_ (0.6 mM) for 6 h. Cell death was measured by PI staining (red) with DAPI nuclear staining (Scale bar, 100 μm). Quantification of propidium iodide imaging. Data shown are the mean ± SD (*n* = 5). Statistical significance was tested by one-way ANOVA, followed by Tukey–Kramer test; ***p < 0.001 (vs. ctrl), ###*p* < 0.001 [vs. YM435 (–)]. +: 50 μM, ++: 100 μM. **H** Cultured brain slice was treated with the indicated concentrations of YM435 and Aβ (25-35; 10 μM) for 60 h, Cell death was measured by PI staining (red) with DAPI nuclear staining (Scale bar, 100 μm). Quantification of propidium iodide imaging. Data shown are the mean ± SD (*n* = 5). Statistical significance was tested by one-way ANOVA, followed by Tukey–Kramer test; ***p < 0.001 (vs. ctrl), ###*p* < 0.001 [vs. YM435 (–)]. +: 50 μM, ++: 100 μM. All data are representative of at least three independent experiments.

It is known that neuronal damage and death triggered by oxidative stress are major causes of neurodegenerative diseases. Therefore, the antioxidant activity of YM435 may be potentially effective for prevention of neurodegenerative diseases triggered by neuronal damage and death. We therefore investigated the inhibitory effect of YM435 in neuronal cells. As shown in Fig. 2A, YM435 clearly inhibited the reduction of cell viability induced by H_2_O_2_ to a similar extent as the PARP-1 inhibitor DPQ or pamiparib treatment in SH-SY5Y cultured neural cells. In addition, similar results were seen in PC12 cells (Figs. 2B and 2C). We next examined whether YM435 can cross the blood-brain barrier (BBB) to evaluate the neuronal protective effect of YM435 in vivo. However, brain concentrations of YM435 were much lower than veliparib that can cross the BBB [9], when 1 mM YM435 or veliparib dissolved in PBS was infused into the jugular vein of C57BL mice for 1 hour (Fig. 2D). We therefore examined the neuroprotective effects of YM435 ex vivo to perform analysis under conditions closer to those of living organisms. In mouse brain slices, propidium iodide (PI) staining revealed that rucaparib, and antioxidants such as propyl gallate and *N*-acetylcysteine significantly reduced PI-positive (dead) cells induced by H_2_O_2_, suggesting that H_2_O_2_ induce oxidative stress-mediated parthanatos in mouse brain slices (Fig. 2E and F). Interestingly, we found that YM435 clearly inhibited H_2_O_2_-induced parthanatos in mouse brain slices (Fig. 2G). Moreover, YM435 successfully prevented oxidative stress-mediated cell death induced by amyloid β-peptide (25-35) that shows a cytotoxic effect on neuronal cells, which is associated with the pathogenesis of Alzheimer’s disease (Fig. 2H) [38, 39]. Thus, YM435 cannot cross the BBB, but its antioxidant activity is effective in ex vivo.

### YM435 suppresses parthanatos independently of its antioxidant activity

**Fig. 3:**
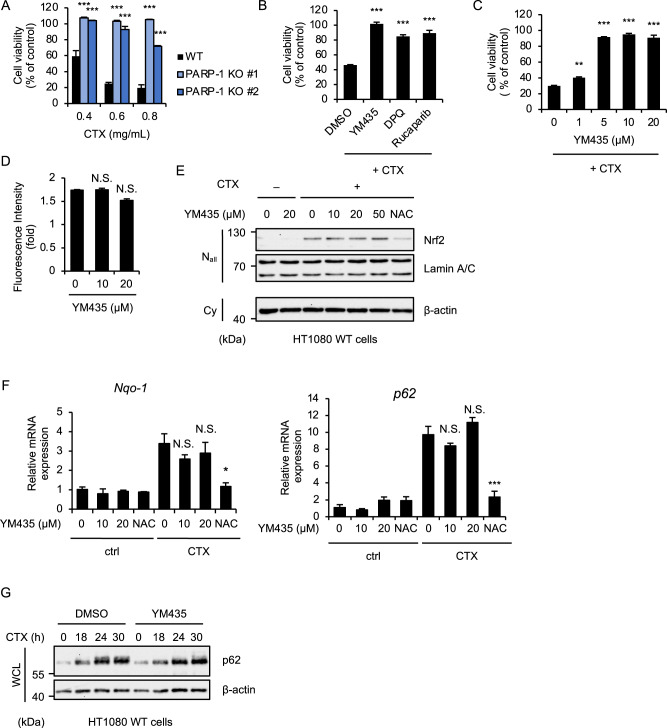
YM435 suppresses parthanatos independently of its antioxidant activity. **A** WT and PARP-1 KO HT1080 cells were treated with the indicated concentrations of CTX for 36 h. Cell viability was measured by PMS/MTS assay. Data shown are the mean ± SD (*n* = 3). Statistical significance was tested by one-way ANOVA, followed by Tukey–Kramer test; ***p < 0.001 (vs. WT). **B** HT1080 cells were treated with YM435 (20 μM), DPQ (30 μM), Rucaparib (1 μM) and CTX (0.6 mg/mL) for 36 h. Cell viability was measured by PMS/MTS assay. Data shown are the mean ± SD (*n* = 3). Statistical significance was tested by one-way ANOVA, followed by Tukey–Kramer test; ***p < 0.001, (vs. DMSO). **C** HT1080 cells were treated with the indicated concentrations YM435 and CTX (0.6 mg/mL) for 36 h. Cell viability was measured by PMS/MTS assay. Data shown are the mean ± SD (*n* = 3). Statistical significance was tested by one-way ANOVA, followed by Tukey–Kramer test; **p < 0.01, ***p < 0.001 (vs. DMSO). **D** HT1080 cells were treated with the indicated concentrations YM435 and CTX (0.6 mg/mL) for 28 h, and then incubated with DCFH-DA (10 μM) for 30 min. Quantification of ROS was calculated by detecting the fluorescence intensity of DCFH-DA. Data shown are the mean ± SD (*n* = 3). Statistical significance was tested by one-way ANOVA, followed by Tukey–Kramer test; N.S.: not significant [vs. YM435 (0 μM)]. **E** HT1080 cells were treated with the indicated concentrations YM435, NAC (2 mM) and CTX (0.8 mg/mL) for 24 h. The nuclear and cytoplasm extracts were subjected to immunoblotting with the indicated antibodies. **F** HT1080 cells were treated with the indicated concentrations YM435, NAC (2 mM) and CTX (0.8 mg/mL) for 24 h, and then the mRNA levels were measured by quantitative real-time PCR. Data shown are the mean ± SD (*n* = 3). Statistical significance was tested by one-way ANOVA, followed by Tukey–Kramer test; ***p < 0.001, *p < 0.05, N.S.: not significant [vs. YM435 (0 μM)]. **G** HT1080 cells were treated with YM435 (20 μM) and CTX (1.0 mg/mL) for the indicated periods. Cell lysates were subjected to immunoblotting with the indicated antibodies. All data are representative of at least three independent experiments.

We next confirmed that YM435 is effective for parthanatos induced by cefotaxime (CTX), a third-generation cephalosporin antibiotic that promotes reactive oxygen species (ROS) generation [40, 41], that particularly triggers parthanatos mediated by the p62-based ALIS [16]. As shown in Figs. 3A and 3B, CTX-induced parthanatos was clearly inhibited by PARP-1 knockout, and the PARP-1 inhibitors as previously described [17]. Interestingly, we noticed that YM435 inhibits parthanatos induced by CTX with much lower concentrations (Fig. 3C), when compared with H_2_O_2_ (Fig. 1D). Moreover, the lower concentrations of YM435 (10 and 20 µM) did not affect CTX-induced ROS generation (Fig. 3D). Our previous studies have demonstrated that CTX-induced ROS generation causes the NF-E2-related factor 2 (Nrf2) activation, which upregulates quinone acceptor oxidoreductase 1 (Nqo1), a well-known target of Nrf2, and promotes the accumulation of p62 protein [41]. Consistent with the observation that YM435 did not affect CTX-induced ROS generation, the nuclear translocation of Nrf2 induced by CTX was not affected by the YM435 treatment (Fig. 3E). Moreover, the induction of Nqo1 and p62, and the accumulation of p62 protein were not affected by the YM435 treatment (Fig. 3F and G). Collectively, these results suggest that YM435 suppresses CTX-induced parthanatos independently of its antioxidant activity.

### YM435 reduces aggregability of the p62-based ALIS

**Fig. 4:**
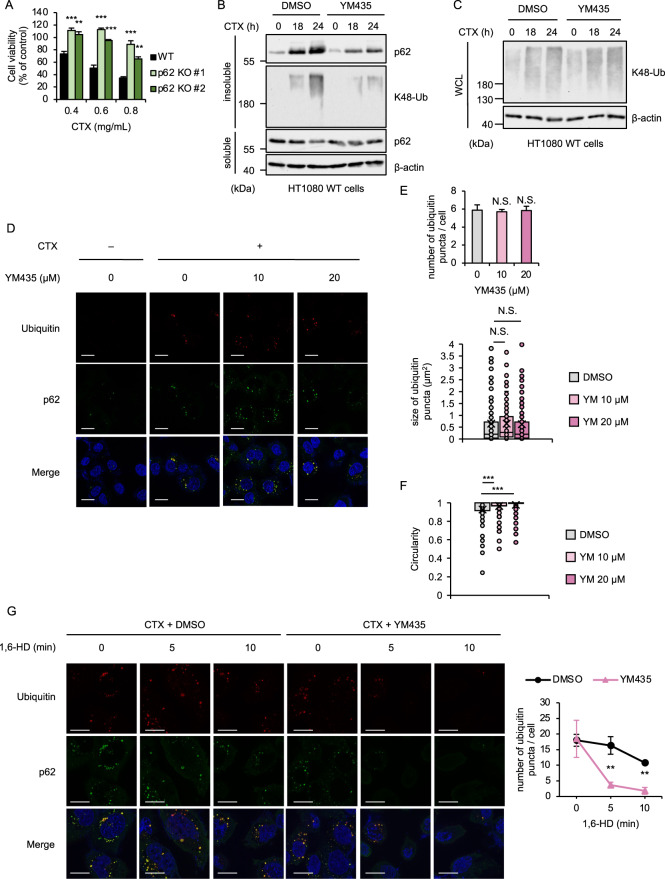
YM435 reduces aggregability of the p62-based ALIS. **A** WT and p62 KO HT1080 cells were treated with the indicated concentrations of CTX for 36 h. Cell viability was measured by PMS/MTS assay. Data shown are the mean ± SD (*n* = 3). Statistical significance was tested by one-way ANOVA, followed by Tukey–Kramer test; **p < 0.01, ***p < 0.001 (vs. WT). **B** HT1080 cells were treated with YM435 (20 μM) and CTX (1.0 mg/mL) for the indicated periods, and then the detergent-soluble and detergent-insoluble fractions were subjected to immunoblotting with the indicated antibodies. **C** HT1080 cells were treated with YM435 (20 μM) and CTX (1.0 mg/mL) for the indicated periods, and then the cell lysates were subjected to immunoblotting with the indicated antibodies. **D** HT1080 cells were treated with the indicated concentrations YM435 and CTX (0.8 mg/mL) for 24 h, and then performed immunofluorescence staining with the indicated antibody, and DAPI nuclear staining (Scale bar, 20 μm). **E** Quantification of number and size of the ubiquitin puncta using imageJ. Data shown are the mean ± SD (number; *n* = 3, size; 0 μM: *n* = 176, 10 μM: *n* = 201, 20 μM: *n* = 177). Statistical significance was tested by Student’s t test; N.S.: not significant [vs. YM435 (0 μM)]. **F** Quantification of circularity of the ubiquitin puncta using imageJ. Data shown are the mean ± SD (0 μM: n = 176, 10 μM: *n* = 201, 20 μM: *n* = 177). Statistical significance was tested by Student’s t test; ***p < 0.001 [vs. YM435 (0 μM)]. **G** HT1080 cells were treated with YM435 (20 μM) and CTX (0.8 mg/mL) for 30 h and 1,6-Hexanediol (1,6-HD) for the indicated periods, and then performed immunofluorescence staining with the indicated antibody, and DAPI nuclear staining (Scale bar, 20 μm). Quantification of number of the ubiquitin puncta using imageJ. Data shown are the mean ± SD (*n* = 3). Statistical significance was tested by Student’s t test; **p < 0.01 (vs. DMSO). All data are representative of at least three independent experiments.

We next examined how YM435 suppresses CTX-induced parthanatos without affecting the ROS generation. We therefore examined whether YM435 affects the formation of the p62-based ALIS, since CTX-induced parthanatos is mediated by the p62-based ALIS that is biochemically depicted as the accumulation of p62 and K48-linked polyubiquitinated proteins in the Triton-X-insoluble fraction [16]. Therefore, p62 is required for CTX-induced parthanatos as previously described (Fig. 4A) [16]. YM435 apparently reduced the accumulation of p62 and K48-linked polyubiquitinated proteins in the Triton-X-insoluble fraction (Fig. 4B), without changing the total amount of the p62 and polyubiquitinated proteins (Figs. 3G and 4C), suggesting the possibility that YM435 suppresses parthanatos by preventing the ALIS formation. On the other hand, the p62-ALIS is microscopically observed as costained puncta of p62 and K48-linked ubiquitin [16]. Given that YM435 inhibits the ALIS formation, the fluorescent puncta of p62 and K48-linked ubiquitin are reduced by the YM435 treatment. However, both the number and size of the fluorescent puncta of p62 and K48-linked ubiquitin were not affected by the YM435 treatment (Figs. 4D and 4E). To explain the discrepancy between the biochemical and microscopic analyses, we speculated that YM435 increases the hydrophilicity of the p62-based ALIS rather than inhibition of its formation. Of note, recent evidence has demonstrated that the circularity of aggregates or liquid droplets reflects its biophysical properties, such as liquidity and aggregability, which prompted us to test the circularity of the p62-based ALIS [42]. Interestingly, it turned out that YM435 significantly increased the circularity of the p62-based ALIS, meaning that YM435 increases the liquidity of the ALIS (Fig. 4F). Moreover, we found that 1,6-Hexanediol (1,6-HD), an organic compound that dissolves liquid droplet, can disaggregate the p62-based ALIS in much shorter time periods (5-10 min), when YM435 was co-treated (Fig. 4G). Considering that YM435 prevented the accumulation of p62 and K48-linked polyubiquitinated proteins in the Triton-X-insoluble fraction, YM435 appears to decrease the aggregability of the p62-based ALIS by increasing its solubility (Fig. 4G).

### Aggregability of the p62-based ALIS determines the sensitivity to parthanatos

**Fig. 5:**
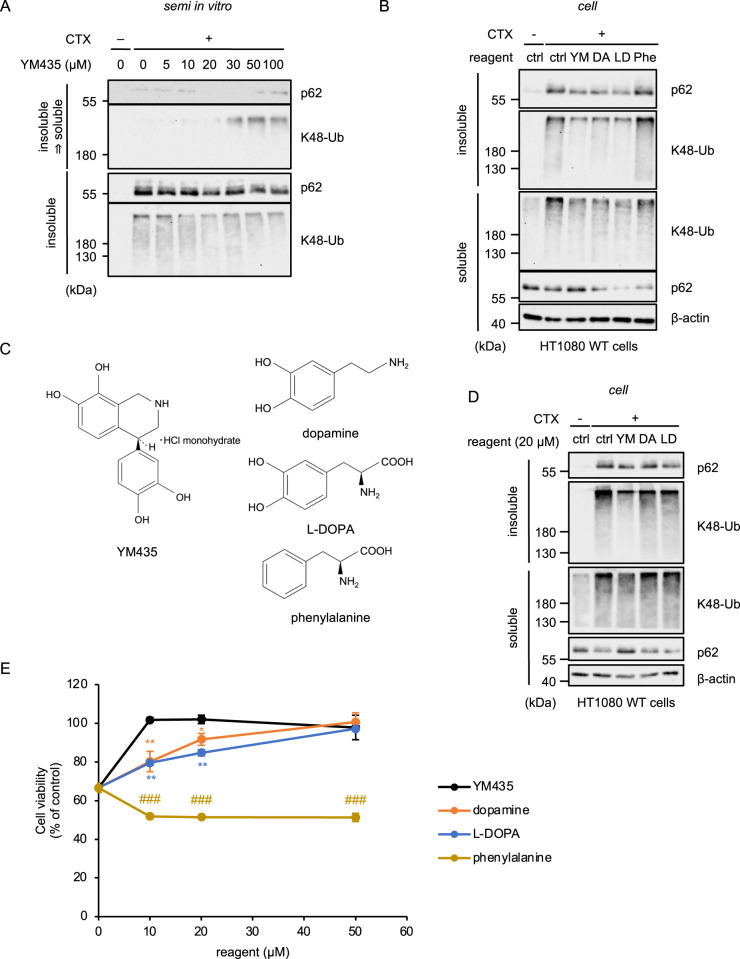
Aggregability of the p62-based ALIS determines the sensitivity to parthanatos. **A** HT1080 cells were treated with CTX (0.8 mg/mL) for 28 h, and then the detergent-insoluble fraction, and YM435-soluble fraction redissolved by YM435 were subjected to immunoblotting with the indicated antibodies. **B** HT1080 cells were treated with CTX (0.8 mg/mL), with YM435 (20 μM), dopamine (DA; 100 μM), L-DOPA (LD; 100 μM), or phenylalanine (Phe; 100 μM) for 28 h,　and then the detergent-soluble and detergent-insoluble fractions were subjected to immunoblotting with the indicated antibodies. **C** Structural formula of the compound used in Fig. 5. **D** HT1080 cells were treated with CTX (0.8 mg/mL), with YM435 (20 μM), dopamine (DA; 20 μM), or L-DOPA (LD; 20 μM) for 28 h,　and then the detergent-soluble and detergent-insoluble fractions were subjected to immunoblotting with the indicated antibodies. **E** HT1080 cells were treated with CTX (0.8 mg/mL), with the indicated concentration of YM435, dopamine, L-DOPA, or phenylalanine for 36 h. Cell viability was measured by PMS/MTS assay. Data shown are the mean ± SD (*n* = 3). Statistical significance was tested by one-way ANOVA, followed by Tukey–Kramer test; **p < 0.01, *p < 0.05 (vs. YM435), ###*p* < 0.001 (vs. L-DOPA). All data are representative of at least three independent experiments.

**Fig. 6:**
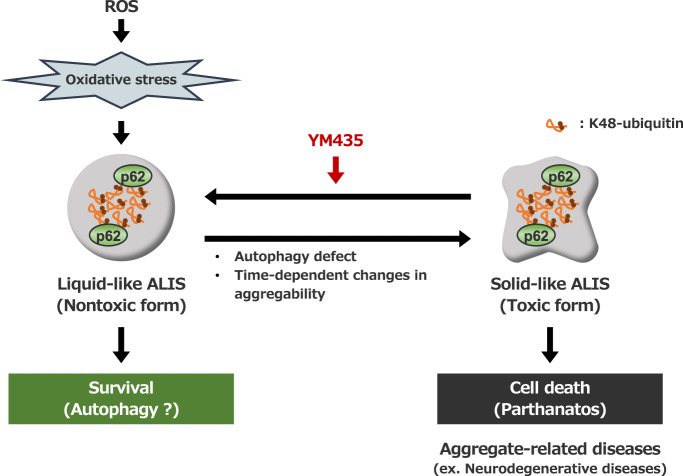
Schematic model to explain our study. The excessive accumulation of ROS initiates oxidative stress, leading to the formation of the p62-based ALIS. In the case of the p62-based ALIS with higher liquidity, the p62-based ALIS is basically nontoxic, and may be degraded by the autophagic processes. On the other hand, the p62-based ALIS with lower liquidity and higher aggregability display cytotoxicity through the induction of parthanatos. The D1 dopamine receptor agonist YM435 reduces aggregability of the p62-based ALIS, and change the cytotoxic ALIS to the nontoxic ALIS. Therefore, YM435 serves as an inhibitor of parthanatos induced by the p62-based ALIS.

We next examined whether the solubilizing effect of YM435 on the p62-based ALIS is brought about by the structural features of YM435 or signaling mechanisms mediated by D1 dopamine receptor. Interestingly, in vitro disaggregation assay revealed that YM435 can dissolve the ubiquitinated proteins on the p62-based ALIS purified from CTX-treated HT1080 cells in vitro, suggesting that YM435 can directly solubilize the ubiquitinated proteins on the p62-baced ALIS independently of D1 dopamine receptor signaling (Fig. 5A). Therefore, we paid attention to the molecular structure of YM435. Interestingly, we found that dopamine and L-DOPA, a dopamine precursor, but not phenylalanine also could disaggregate the p62-based ALIS (Fig. 5B). As shown in Fig. 5C, two hydroxy groups on the benzene ring are common structures among YM435, dopamine and L-DOPA, a dopamine precursor. On the other hand, phenylalanine that has a structure in which two hydroxyl groups are removed from L-DOPA failed to dissolve the ALIS (Fig. 5B). This observation indicates that two hydroxy groups on the benzene ring of the dopaminergic agonists are involved in their solubilizing effect. Finally, we tested whether the solubilizing effect of the dopaminergic agonists contribute to suppress CTX-induced parthanatos mediated by the p62-based ALIS. As shown in Fig. 5D, only YM435 successfully dissolved the ALIS when lower concentrations of reagents (20 μM) were treated. Consistent with this observation, YM435 sufficiently inhibited parthanatos at the lower range (10-20 μM) (Fig. 5E). In addition, 50 μM of dopamine and L-DOPA but not phenylalanine clearly inhibited parthanatos (Fig. 5E). Thus, these observations suggest that aggregability of the p62-based ALIS determines the sensitivity to parthanatos mediated by the p62-based ALIS (Fig. 6).

## Discussion

In this study, we demonstrated that YM435 suppresses parthanatos through the two distinct functions: the antioxidant and disaggregating agent. Furthermore, we also demonstrated that the catecholamines such as dopamine and L-DOPA can disaggregate the p62-based ALIS. Considering that phenylalanine that has a structure in which two hydroxyl groups are removed from L-DOPA failed to disaggregate the p62-based ALIS, it is likely that two hydroxy groups on the benzene ring of YM435 are important for the disaggregation of the p62-based ALIS. Of note, the disaggregating effect of YM435 was observed at lower concentrations than the antioxidant effect of YM435, indicating that YM435 may be useful as a disaggregating agent rather than an antioxidant (Figs. 1D and 3C). As previous studies have shown, these catecholamines can disaggregate the amyloid aggregates composed of amyloid β or α-synuclein [24, 43]. In addition, polyubiquitin and polyubiquitinated proteins have been reported to adopt amyloid-like structures [44]. Therefore, YM435 may disaggregate the p62-based ALIS by acting on the amyloid-like structures of ubiquitin proteins. If so, since the abilities of YM435 to disaggregate the p62-based ALIS and to protect cells from parthanatos were more potent than dopamine and L-DOPA as shown in 5D and 5E, YM435 may be more effective in disaggregating amyloid-like structures. On the other hand, we found that YM435 suppresses cell death induced by amyloid β-peptide (25-35) in brain tissue (Fig. 2H). It has been reported that amyloid β-peptide (25-35) not only induces ROS generation but also forms aggregates [45]. Therefore, further analysis is required to determine whether YM435’s antioxidant ability of or disaggregating effect of YM435 is important for suppressing amyloid β-peptide (25-35)-induced cell death. In any case, the cytoprotective activity of YM435 may be applicable to neuroprotective therapies, although YM435 does not appear to cross the BBB as shown in Fig. 2D. Thus, further studies on drug delivery system will determine whether YM435 is appropriate as a therapeutic agent.

On the other hand, this study regarding YM435 shows that sensitivity to parthanatos is determined by aggregability of the p62-based ALIS. p62 is known to form liquid droplets with ubiquitinated proteins, which serves as the origin of selective autophagy, whereas undegraded liquid droplets tend to have solid-like properties with its maturation [46, 47]. Therefore, accumulation of the liquid droplets with solid-like properties (higher aggregability) seems to mean that autophagic degradation is disturbed, or droplet formation is accelerated beyond the control of autophagy. Indeed, in the presence of lysosomal stresses that disturb autophagy, p62 forms highly hydrophobic liquid droplets [48]. Since these cellular conditions usually arise in pathologic and stress condition, the aggregability of the p62 liquid droplet may serve as a barometer of cellular clearance. Therefore, it seems to be reasonable that the induction of parthanatos is determined by the aggregability of the p62-based ALIS. Taken together, our investigation of the cellular response of YM435 brought about a better understanding of the biological significance of parthanatos that the p62-based ALIS with higher aggregability caused by hypoactivity of cellular clearance trigger cell death. Although it is still unknown how the p62-based ALIS with higher aggregability initiate parthanatos, our results uncovered novel aspect related to cell life and death, which may new insight into the pathogenesis of the stress-induced tissue damage and diseases.

## Materials and Methods

### Cell lines and reagent

HT1080 cells were from JCRB Cell Bank (Japanese Collection of Research Bioresources Cell Bank) [49]. PC12 and SH-SY5Y cells were provided by RIKEN BRC (Tsukuba, Japan) [50, 51]. HT1080 cells were grown in Dulbecco’s Modified Eagle Medium (DMEM), 5% heat-inactivated fetal bovine serum (FBS), and 1% penicillin-streptomycin solution, at 37 °C under a 5% CO_2_ atmosphere. SH-SY5Y cells and PC12 cells were grown in Dulbecco’s Modified Eagle Medium (DMEM), 10% heat-inactivated fetal bovine serum (FBS), and 1% penicillin-streptomycin solution, at 37 °C under a 5% CO_2_ atmosphere. All cell lines are confirmed to be mycoplasma-free. The following reagents were obtained from commercial sources; cefotaxime (CTX), hydrogen peroxide (H_2_O_2_), N-Acetyl-L-cysteine (NAC), DPQ (Wako, Osaka, Japan), Rucaparib, Pamiparib, Niraparib, veliparib, E7449 (Selleck, Houston, TX, USA), Propyl Gallate (PG; Sigma, St. Louis, MO, USA), 1,6-Hexanediol (1,6-HD; Tokyo Chemical Industry Co., Ltd., Tokyo, Japan), Dopamine hydrochloride, L-DOPA, L-(-)-Phenylalanine (Nacalai tesque, Kyoto, Japan). YM435 was synthesized at Astellas Pharma Inc. The antibodies used were against Nrf2, PARP-1, ubiquitin (P4D1), Lamin A/C, Fibrillarin, α-Tubulin (Santa Cruz, Dallas, TX, USA), K48-linked ubiquitin, Mono/Poly ADP-ribose (PAR) (Cell signaling, Danvers, MA, USA), p62 (MBL, Tokyo, Japan; for IF), p62 (BD, New Jersey, USA; for IB), β-actin (Wako).

### Generation of knockout cell lines

PARP-1 and p62 knockout (KO) cells were established by using the CRISPR/Cas9 system, and characterized as described previously [17]. Guide RNAs (gRNAs) were designed to target a region in the exon 1 of PARP-1 gene (5’-GAGTCGAGTACGCCAAGAGC-3’), and that in the exon 3 of p62 gene (5’-AGACTACGACTTGTGTAGCG-3’) using CRISPRdirect. Then, gRNA-encoding oligonucleotide was cloned into lenti-CRISPRv2 plasmid (addgene, Watertown, MA, USA), and the plasmid was transfected with HEK293A cells with an envelope plasmid pVSV-G and a packaging plasmid psPAX2. The supernatants were used to infect HT1080 cells, and then infected cells were cloned by limiting dilution to obtain 100% efficiency in the presence of puromycin. To check the mutations of PARP-1 and p62 in cloned cells, genomic sequence, including the target region was analyzed by PCR using extracted DNA from each clone as a template, and the following primers were used. For determination of PARP-1 mutation; 5’-GCATCAGCAATCTATCAG-3’ and 5’-CTTCCCGGACACAGTTAA-3’. For determination of p62 mutation; 5′-GAGGACTTTAGGGGGTCCCA-3′ and 5′-AGGAATTAGCAGAGCGGCAG-3′. Cloned cell with mutations near the target sequence were established as PARP-1 or p62 KO cells (#1 or #2).

### Nuclear/chromatin extraction

Nuclear/chromatin extraction was performed as described previously with minor modifications [52]. Briefly, the cells were washed with phosphate-buffered saline (PBS) and pelleted at 1500x *g* in an eppendorf tube at 4 °C for 3 min. Pellets were resuspended by gentle pipetting in ice-cold E1 buffer [50 mM Hepes-KOH (pH 7.5), 100 mM NaCl, 1 mM EDTA pH 8.0, 10% glycerol, 0.5% NP-40, 0.25% triton X-100, 1 mM DTT, and 1% protease inhibitor cocktails (Nacalai Tesque, Kyoto, Japan)], and then centrifuged at 1,100 x *g* at 4°C for 5 min. The supernatant was collected in a fresh tube (cytoplasm fraction; Cy) and the pellet was resuspended by gentle pipetting in the same volume of E1 buffer and centrifuged as before, and the supernatant was discarded twice. The pellets were resuspended by gentle pipetting in ice-cold E2 buffer [10 mM Tris-HCl (pH8.0), 300 mM NaCl, 1 mM EDTA (pH 8.0), 0.5 mM EGTA (pH 8.0), and 1% protease inhibitor cocktails] and centrifuged at 1100x *g* at 4°C for 5 min. The supernatant was collected in a fresh tube (Nucleus fraction; N) and the pellet was resuspended by gentle pipetting in the same volume of E2 buffer and centrifuged as before, and the supernatant was discarded twice. The pellets were lysed in RIPA buffer [25 mM Tris-HCl (pH 7.4), 150 mM NaCl, 1% NP-40, 1% deoxycholate, and 0.1% SDS], supplemented 1% SDS and 0.02% benzonase, and collected in a fresh tube (Chromatin fraction; Ch). All Nucleus fractions; N_all_ is a lysed sample of pellet after sampling in E1 buffer with RIPA buffer supplemented 1% SDS and 0.02% benzonase.

### Immunoblot

Cell extracts were fractionated into detergent-soluble and -insoluble fractions using the 1% Triton X-100 buffer [20 mM Tris-HCl (pH 7.4), 150 mM NaCl, 1% Triton-X100, 10% Glycerol, and 1% protease inhibitor cocktails]. The detergent-insoluble fraction was lysed in RIPA buffer supplemented 1% SDS and 0.02% benzonase. Both the detergent-soluble and -insoluble fractions were subjected to immunoblot analysis as previously described [53].

### in vitro disaggregation assay

The insoluble fraction of the cell extract was dissolved in the 1% Triton X-100 buffer containing each compound, shaken for 30 min, and centrifuged at 15,000 rpm at 4 °C for 15 min. The supernatant was collected in a fresh tube (insoluble → soluble fraction). The pellets were lysed in RIPA buffer supplemented 1% SDS and 0.02% benzonase and collected in a fresh tube (insoluble fraction).

### Colorimetric cell viability assay

Cell viability assay using Cell Titer 96 Cell Proliferation Assay (Promega, Madison, Wisconsin, USA) was performed as described previously [54]. Cells were seeded on 96-well plates. After indicated stimulation or treatment, cell viability was determined according to the manufacturer’s protocol. The absorbance was read at 492 nm using a microplate reader. Data are normalized to control (100%) without stimulus, unless noted otherwise.

### FACS analysis

FACS analysis was performed as described previously [55]. To detect ROS generation, HT1080 cells were stimulated with CTX and then incubated with 2′,7′-dichlorodihydrofluorescein diacetate (DCFH-DA; Wako) for 30 min. Fluorescence intensity was calculated by flow cytometry with the excitation wavelength at 488 nm and the emission wavelength at 580 nm. Fluorescent cells were detected by CytoFLEX (Beckman Coulter, Brea, California, USA).

### Quantitative real-time PCR

Total RNA was isolated by using Sepasol-RNA I Super G (Nacalai Tesque) and reverse transcribed by using High-Capacity cDNA Reverse Transcription Kit (Applied Biosystems) according to the manufacturer’s instructions [56]. Template cDNA was amplified by quantitative real-time PCR with KAPA SYBR FAST qPCR Kits (KAPA Biosystems) according to the manufacturer’s instructions. Primers used for qRT-PCR; 5’-CCGTGGATCCCTTGCAGAGA-3’ and 5’-AGGACCCTTCCGGAGTAAGA-3’ for human *Nqo1*, 5’-ATGGGTCCACCAGGAAACT-3’ and 5’-TGCTCTTCTCCTCTGTGCTG-3’ for human *p62*, 5’-AACAGCCTCAAGATCATCAGC-3’ and 5’-GGATGATGTTCTGGAGAGCC-3’ for human *GAPDH*. Each gene expression levels were normalized to that of *GAPDH*.

### Immunofluorescence staining

HT1080 cells were fixed with 3.7% formaldehyde, permeabilized with 0.5% Triton X-100, blocked with 3% BSA- PBS, and incubated with primary　antibodies (anti-p62, anti-ubiquitin) overnight at 4°C, followed by incubation with secondary antibodies (p62: goat anti-rabbit Alexa Fluor488, ubiquitin: goat anti-mouse Alexa Fluor 555, Invitrogen) for 1 h at room temperature. The immunostained samples were enclosed with Fluoro-KEEPER Antifade Reagent, Non-Hardening Type with DAPI (Nacalai Tesque), and observed using a BZ-X800L fluorescence microscope. For every sample, three images were acquired randomly, and the number, size, and circularity of ubiquitin puncta were measured by ImageJ [57].

### Alkaline Comet assay

The Comet assay was performed according to the protocol of Comet SCGE assay kit (Enzo Life Sciences, Inc., NewYork, USA).　Briefly, HT1080 cells are mixed with agarose in which the cells have been thawed, applied to slides, and allowed to stand at 4°C for 10 minutes. After the agarose has set, the slides were immersed in lysis solution at 4°C for 30 minutes. After that, the slides are immersed in Alkaline solution -NaOH (EDTA 1 mM, pH>13) for 30 minutes at room temperature, then electrophoresed at 25 V for 5 minutes. CyGREEN was added to the slides and stained for 30 minutes, followed by observation under a fluorescence microscope. Comet scores were calculated according to comet tail length (*n* = 25) [37].

### Animal experiment

All mouse pups used in the study were C57BL/6. The parent mice were purchased from CLEA Japan (Shizuoka, Japan). Mouse brain slice cultures were prepared according to the previously reported preparation method [58]. Briefly, 1 h before dissecting the mice, 600 µL of slice culture medium (50% Opti-MEM, 25% Hanks’ Balanced Salt Solution [HBSS], 25% heat-inactivated horse serum, 2 mM L-glutamine, 6.5 mg/mL glucose, and 1% penicillin-streptomycin solution) were added to each well of a 12-well plate, and incubated in 5% CO_2_ at 37°C. The cerebral cortices of the mouse pups (P7) were dissected and immediately placed in ice-cold Dulbecco’s Modified Eagle Medium (DMEM), for 5 min. The cerebral cortices were cut into 350 µm-thick sagittal slices using a McIlwain Tissue Chopper (Mickle Laboratory Engineering, Cambridge, UK) and incubated on ice for 30 min in DMEM. A slice was placed on the culture plate insert of the 12-well plate and maintained in 5% CO_2_ at 37°C. The incubation media were replaced after 24 h and used for after 3 days. Each experiment was started 10 days after slicing. In order to test whether YM435 crosses the BBB, 1 mM YM435 or veliparib dissolved in PBS was infused into the jugular vein of C57BL mice (*n* = 4) for 1 hour (200 μL / hr) under general anesthesia, and then plasma and brain were collected. The concentration of YM435 or veliparib was measured by using LC / MS / MS as previously described [59].

### PI staining

After each stimulation, mouse brain slices were stained with PI (5 µg/mL) in the medium for 1 h. Slices were fixed with 3.7% formaldehyde, and then enclosed with Fluoro-KEEPER Antifade Reagent, Non-Hardening Type with DAPI (Nacalai Tesque), and observed using a BZ-X800L fluorescence microscope. For every slice, five images were acquired randomly, and the number of PI puncta were counted for each image.

### Statistical analysis

The value was expressed as the mean ± standard deviation (S.D.) using Prism software (GraphPad). All experiments were repeated at least three independent times. Two groups were compared using student’s t-test. Multiple-group comparisons were conducted using the one-way ANOVA analysis of variance followed by the Tukey–Kramer test using Prism software (GraphPad). Data were considered significant when *p < 0.05, **p < 0.01, ***p < 0.001.

### Supplementary information

Original Data File

## Data Availability

All data needed to evaluate the conclusions in the paper are present in the paper or the Supplementary Materials.
