# Supplementary material for: Aggregability of the SQSTM1/p62-based aggresome-like induced structures determines the sensitivity to parthanatos
Source: Cell Death Discov. 2024 Feb 12;10:74. doi: 10.1038/s41420-024-01838-2 (PMC10861449; doi:10.1038/s41420-024-01838-2)
Supplement: Supplementary file 1 — Original Data File [file 41420_2024_1838_MOESM1_ESM.pdf]

Full-length and uncropped western blot for Figure 1A

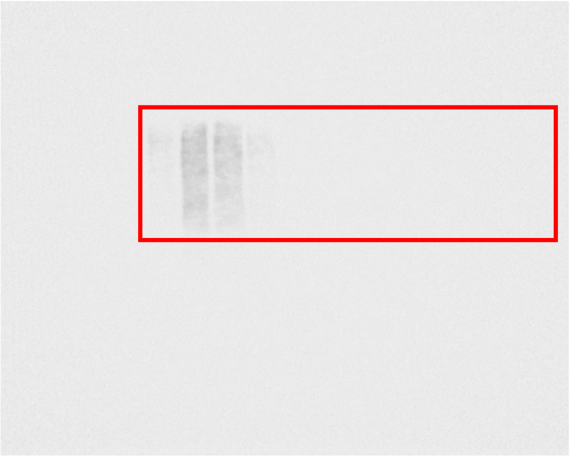

Figure 1A PAR

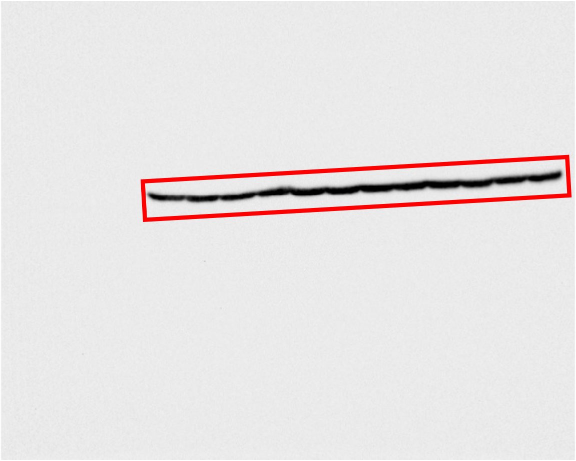

Figure 1A β-actin

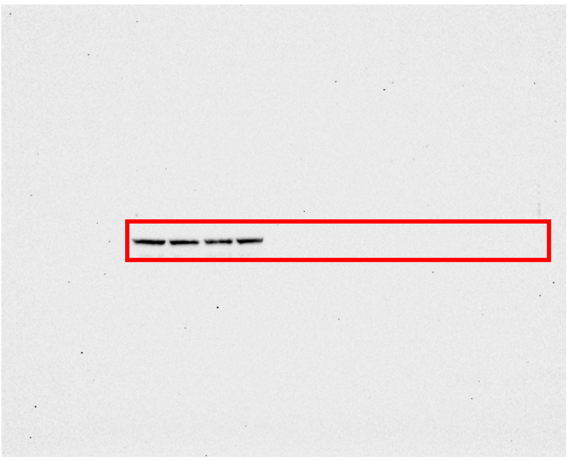

Figure 1A PARP-1

Full-length and uncropped western blot for Figure 1E

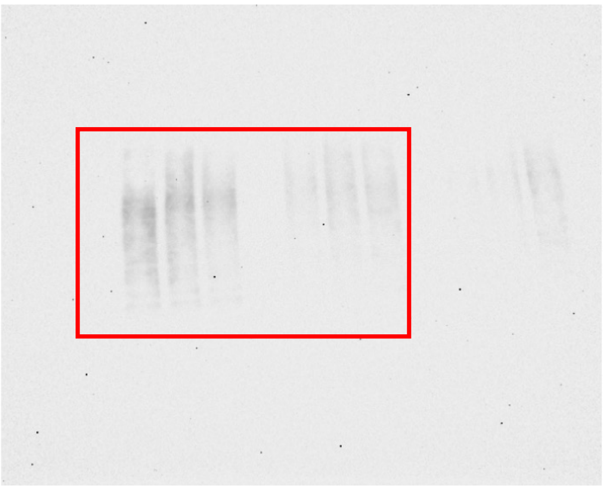

Figure 1E PAR

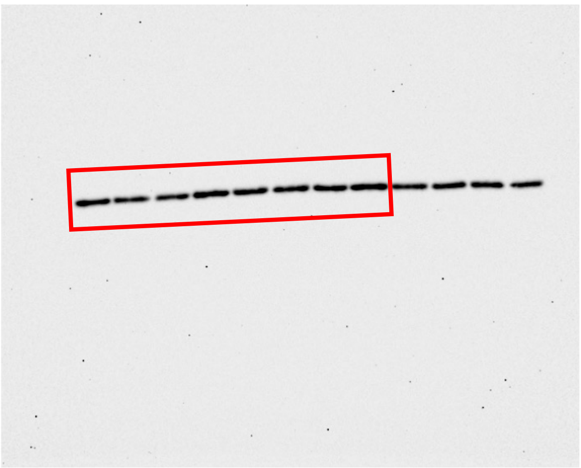

Figure 1E β-actin

Full-length and uncropped western blot for Figure 1F

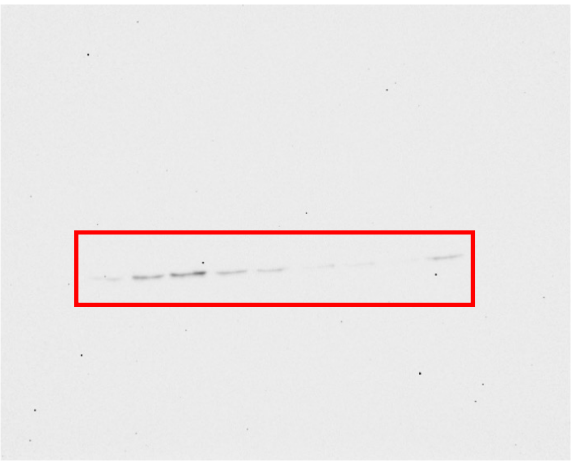

Figure 1F AIF (N_{all})

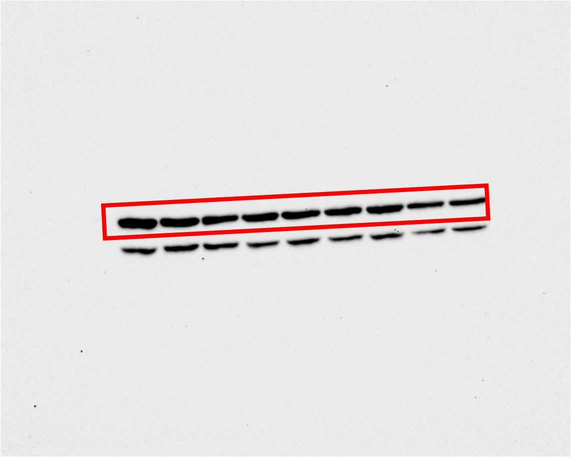

Figure 1F Lamin A/C

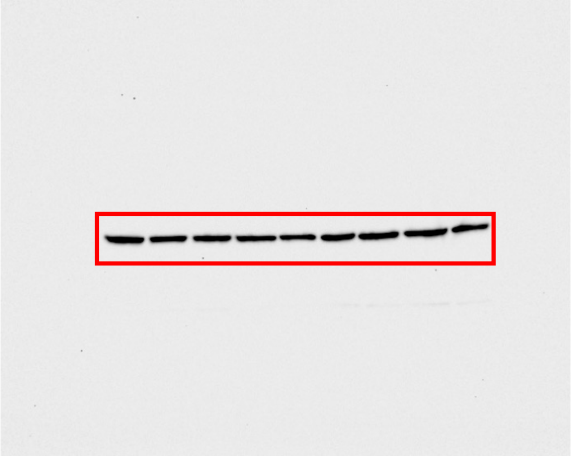

Figure 1F AIF (Cy)

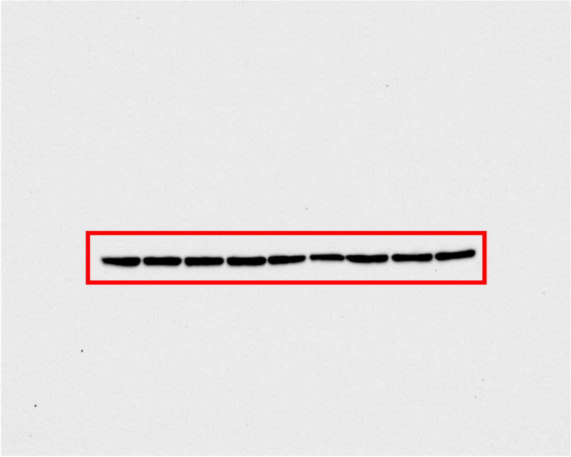

Figure 1F α-Tubulin

Full-length and uncropped western blot for Figure 1G

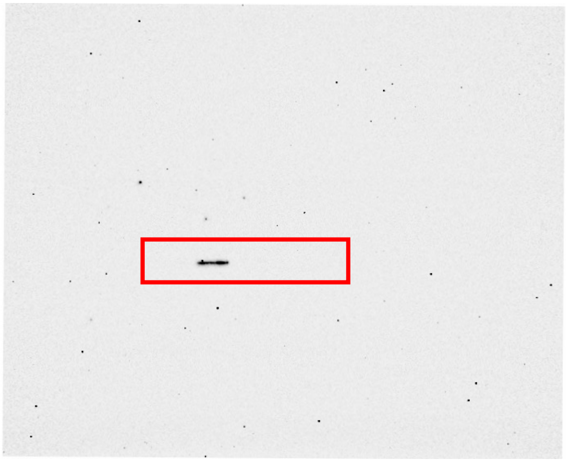

Figure 1G PARP1 (Ch)

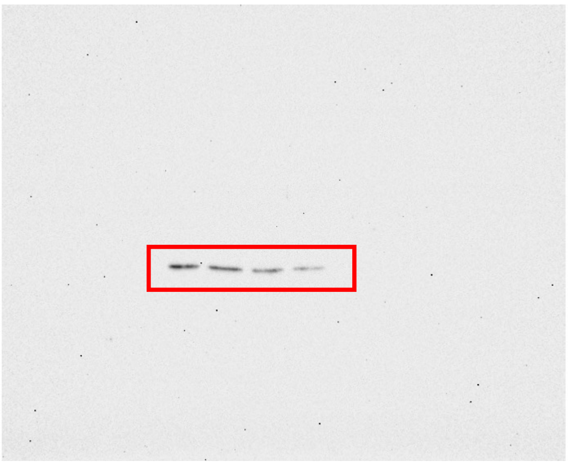

Figure 1G Fibrillarin

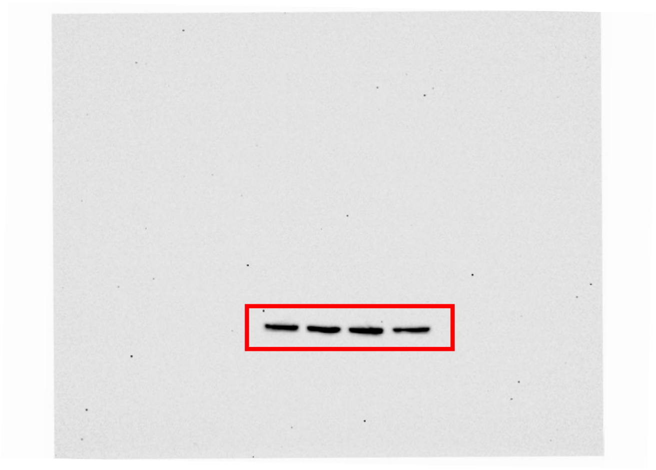

Figure 1G PARP1 (N)

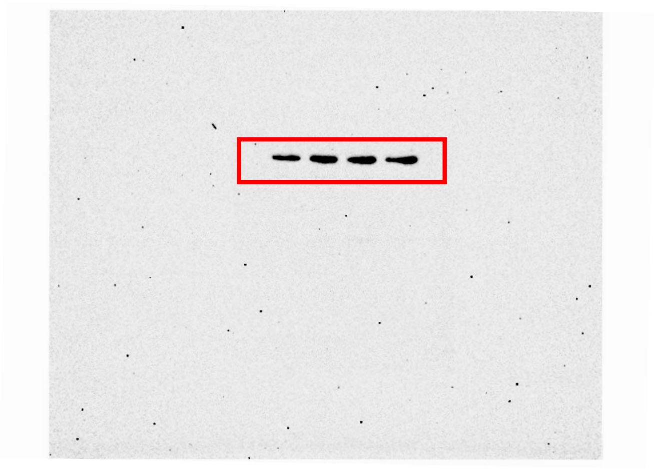

Figure 1G Lamin A

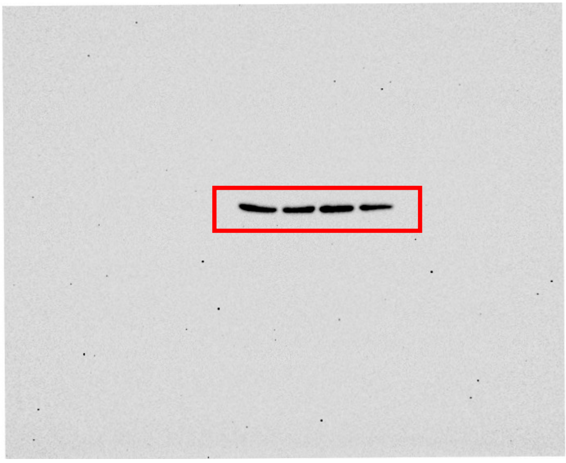

Figure 1G β-actin

Full-length and uncropped western blot for Figure 3E

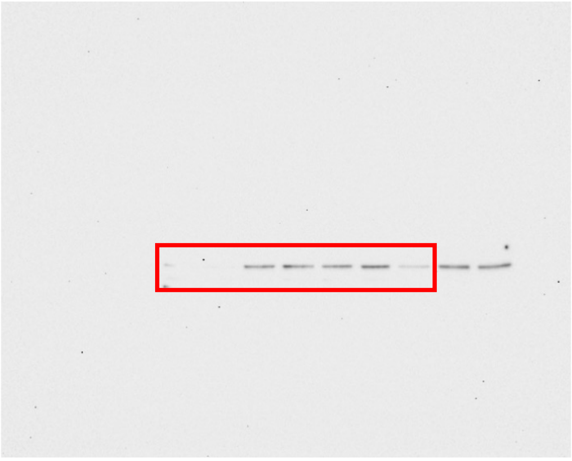

Figure 3E Nrf2

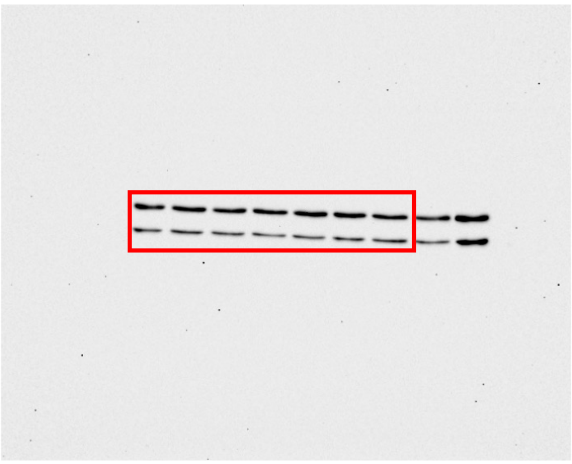

Figure 3E Lamin A/C

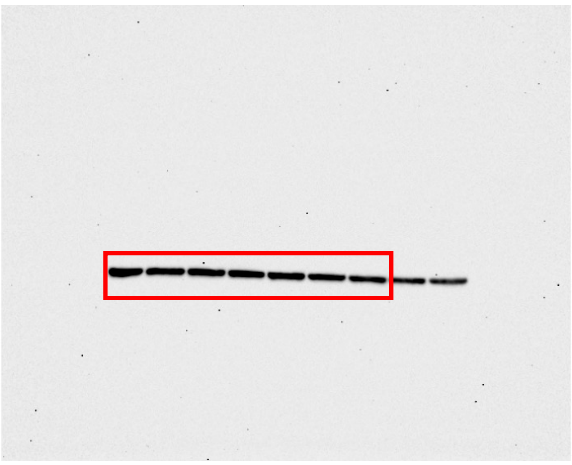

Figure 3E β-actin

Full-length and uncropped western blot for Figure 3G

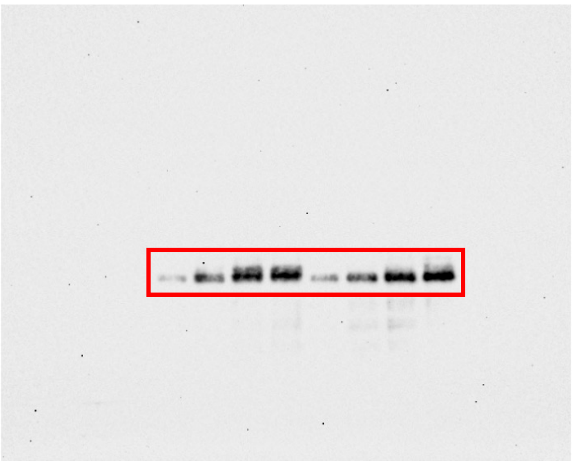

Figure 3G p62

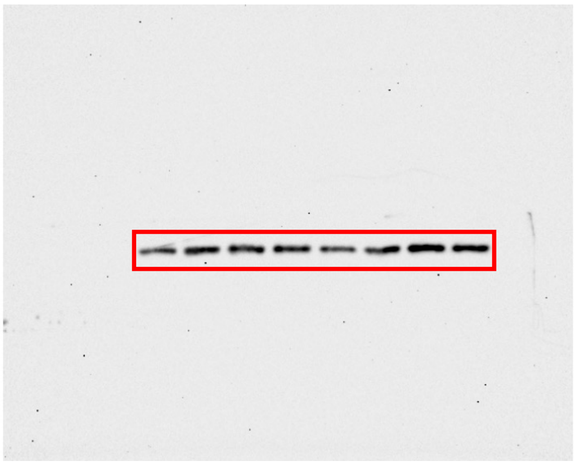

Figure 3G β-actin

Full-length and uncropped western blot for Figure 4A

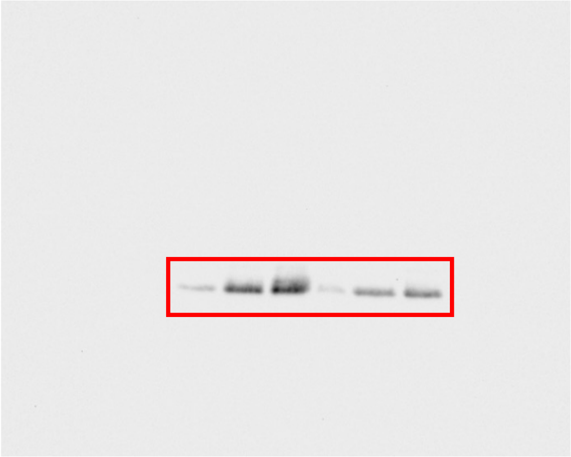

Figure 4A p62 (insoluble)

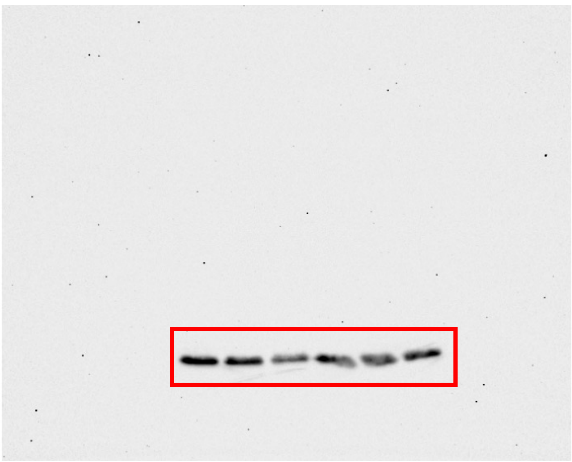

Figure 4A p62 (soluble)

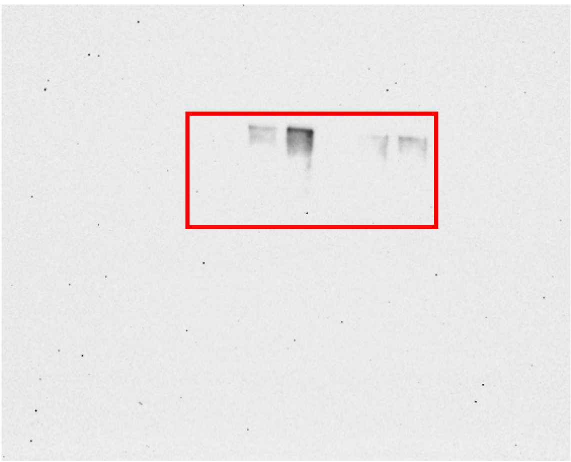

Figure 4A K48-Ub

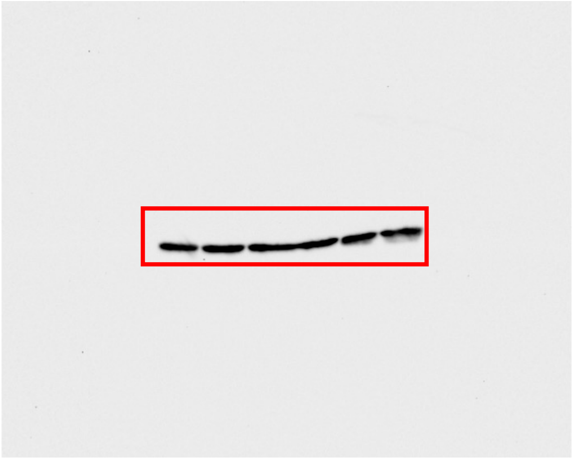

Figure 4A β-actin

Full-length and uncropped western blot for Figure 4B

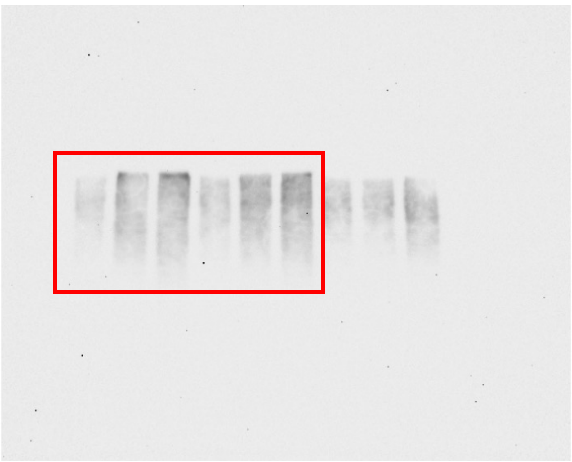

Figure 4B K48-Ub

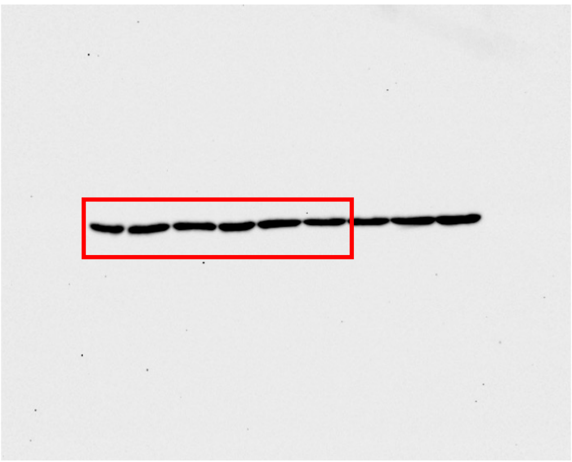

Figure 4B β-actin

Full-length and uncropped western blot for Figure 5A

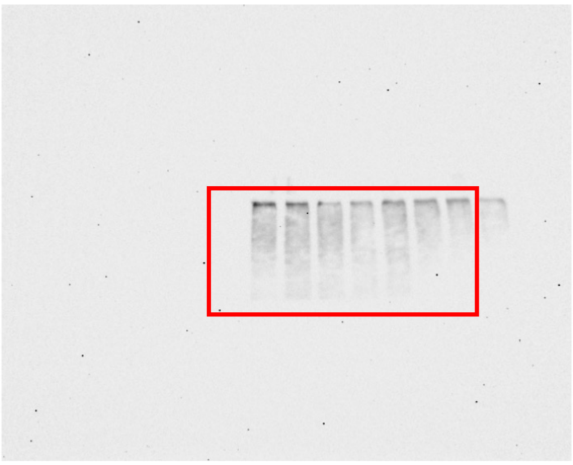

Figure 5A K48-Ub (insoluble)

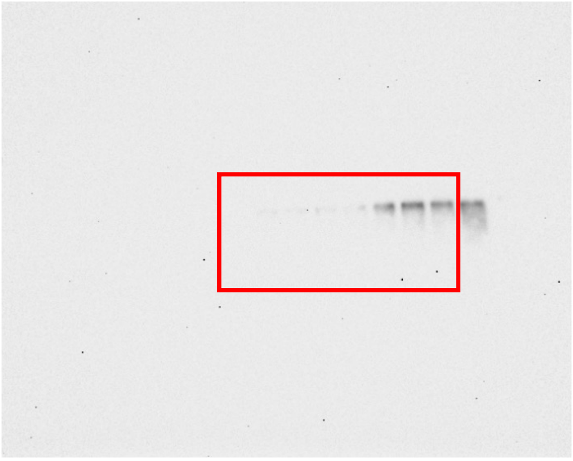

Figure 5A K48-Ub (insoluble ⇒ soluble)

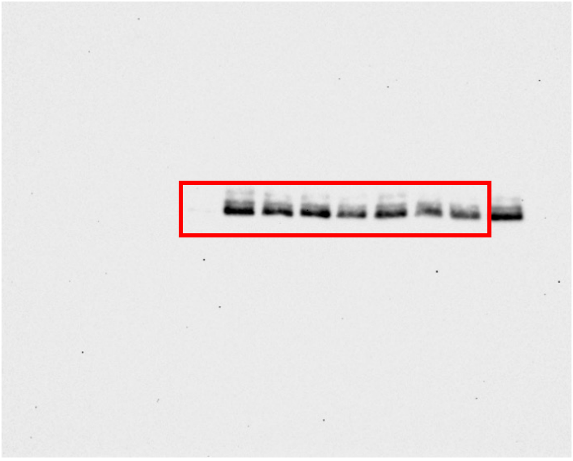

Figure 5A p62 (insoluble)

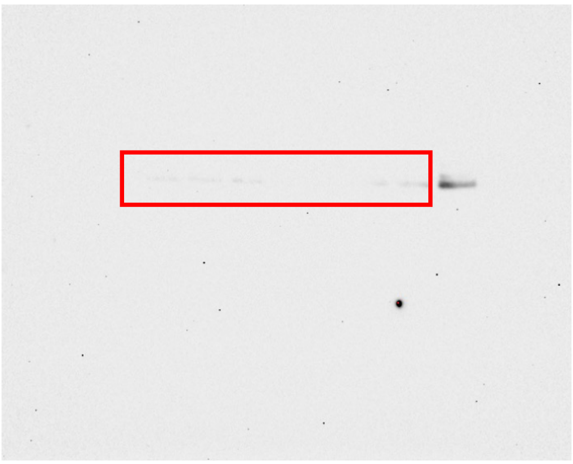

Figure 5A p62 (insoluble ⇒ soluble)

Full-length and uncropped western blot for Figure 5B

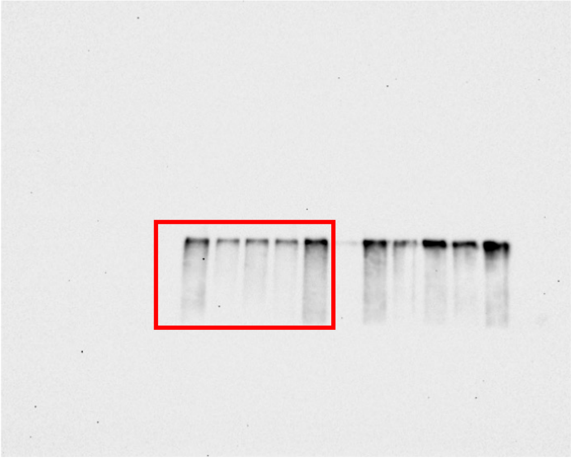

Figure 5B K48-Ub (insoluble)

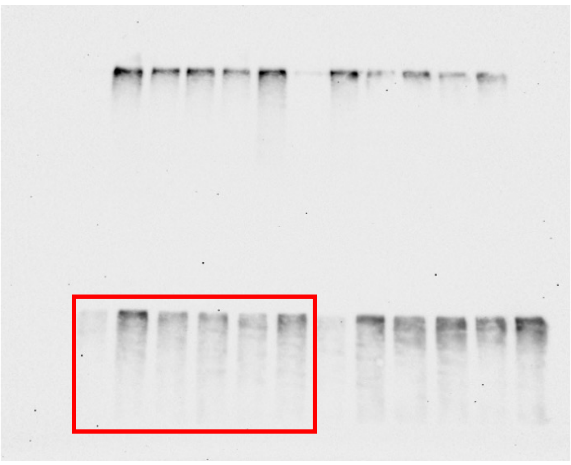

Figure 5B K48-Ub (soluble)

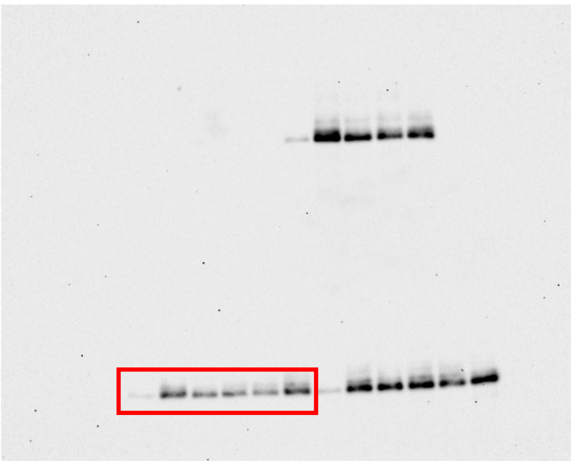

Figure 5B p62 (insoluble)

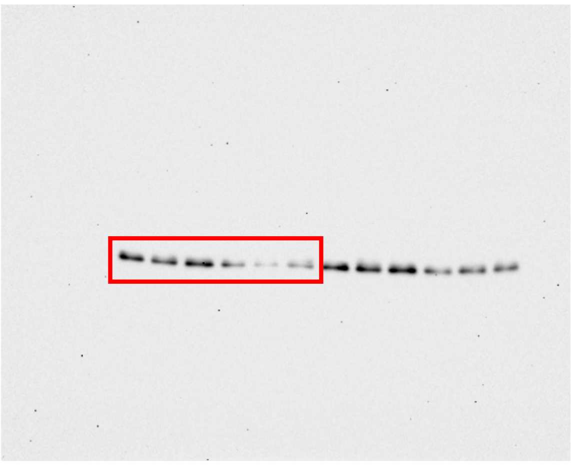

Figure 5B p62 (soluble)

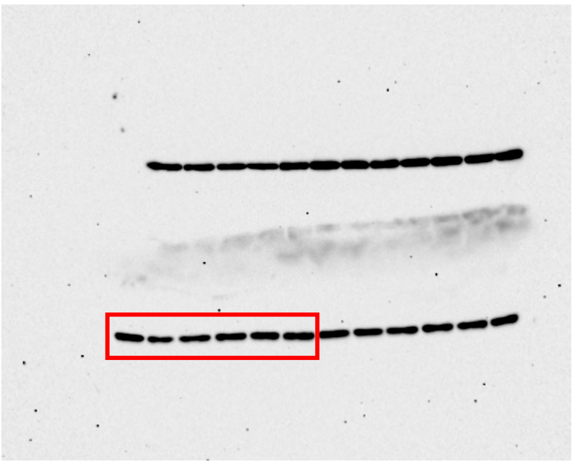

Figure 5B β-actin

Full-length and uncropped western blot for Figure 5D

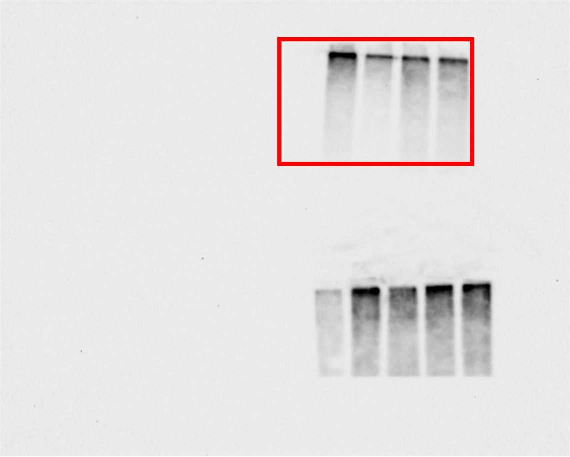

Figure 5D K48-Ub (insoluble)

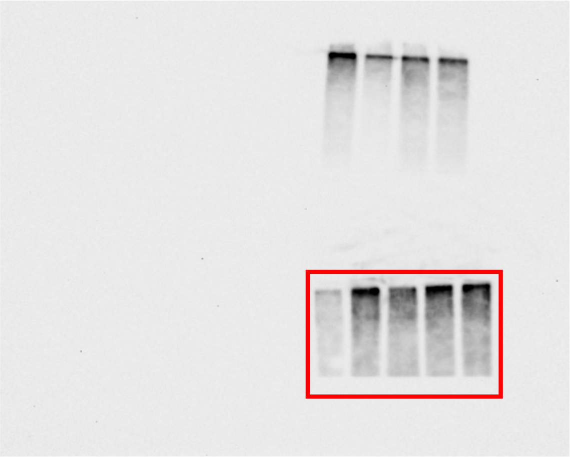

Figure 5D K48-Ub (soluble)

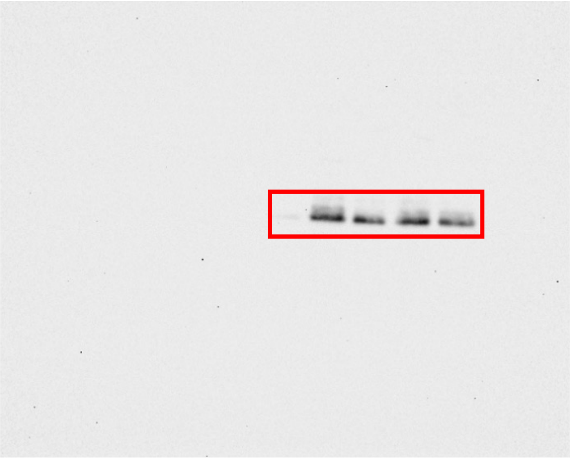

Figure 5D p62 (insoluble)

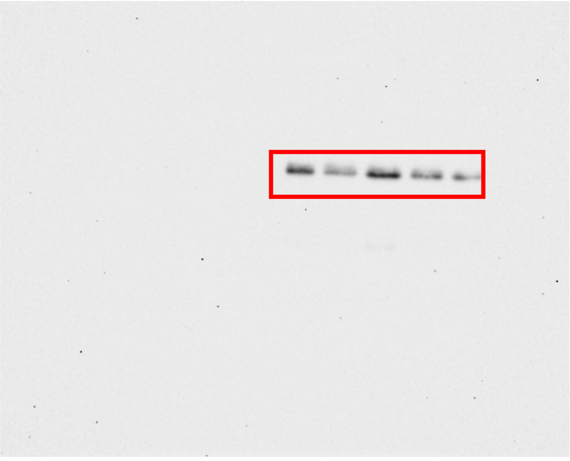

Figure 5D p62 (soluble)

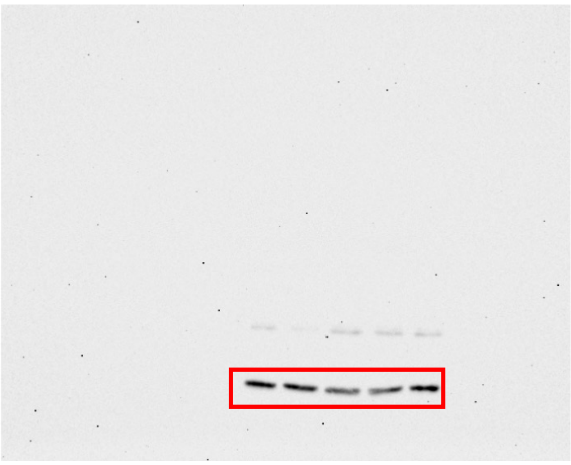

Figure 5D β-actin
